# Iron homeostasis in host and gut bacteria – a complex interrelationship

**DOI:** 10.1080/19490976.2021.1874855

**Published:** 2021-02-04

**Authors:** Yohannes Seyoum, Kaleab Baye, Christèle Humblot

**Affiliations:** a Center for Food Science and Nutrition, College of Natural and Computational Sciences, Addis Ababa University, Addis Ababa, Ethiopia; b QualiSud, Université de Montpellier, Avignon Université, CIRAD, Institut Agro, IRD, Université de la Réunion, Montpellier, France

**Keywords:** Anemia, bacteria, gut, human, iron, metabolism, microbiota, rodent

## Abstract

Iron deficiency is the most frequent nutritional deficiency in the world with an estimated 1.4 billion people affected. The usual way to fight iron deficiency is iron fortification, but this approach is not always effective and can have undesirable side effects including an increase in the growth and virulence of gut bacterial pathogens responsible for diarrhea and gut inflammation. Iron is mainly absorbed in the duodenum and is tightly regulated in mammals. Unabsorbed iron enters the colonic lumen where many microorganisms, referred to as gut microbiota, reside. Iron is essential for these bacteria, and its availability consequently affects this microbial ecosystem. The aim of this review is to provide further insights into the complex relationship between iron and gut microbiota. Given that overcoming anemia caused by iron deficiency is still a challenge today, gut microbiota could help identify more efficient ways to tackle this public health problem.

## Introduction

About 1.4 billion people in the world are estimated to be affected by iron deficiency^1^. Iron is an essential component of hemoglobin in red blood cells and of myoglobin in muscles, which together contain around 60% of total body iron. Iron is also essential for the functioning of cellular mechanisms, including enzymatic processes, DNA synthesis, and generating mitochondrial energy.^2^ However, iron can also be toxic since it catalyzes the production of reactive oxygen species, which can damage lipids, nucleic acids, and proteins.^3^ Therefore, iron homeostasis is tightly regulated in mammals, especially in the duodenum where iron is mainly absorbed.

In the digestive tract, epithelial cells are in contact with a large number of microorganisms, referred to as gut microbiota whose role in health is increasingly recognized today.^4^ The gut microbiota encounters a broad range of concentrations of unabsorbed luminal iron originating from the diet. Iron is essential for bacteria as it functions as a co-factor in iron-containing proteins in redox reactions, metabolic pathways, and electron transport chain mechanisms.^5^ The host’s iron status and dietary iron availability affect this microbial ecosystem.^6–8^ For example, oral administration of iron can enhance the growth and virulence of gut bacterial pathogens resulting in diarrhea and gut inflammation.^9,10^ Iron deficiency also affects microbial composition, strengthening nutritional immunity against pathogenic invaders by restricting access to iron.^11^

One of the ways used to fight iron deficiencies is iron fortification and/or supplementation. However, these programs are not always effective and may have serious side effects.^12–15^ This suggests that micronutrient interventions, like supplementation and fortification, based solely on the prevalence of undernutrition and broad assessments of the prevailing diets are not very effective and in the worst case, may increase the risk of adverse effects. Only a few studies have accounted for host factors including the composition of gut microbiota, which may also influence the response to such interventions.

In general, the bioavailability of iron fortificants varies but is always low since only between 5% and 15% is absorbed in the duodenum while the rest goes to the colon where it becomes available to resident microorganisms. Most studies of the bacterial use of iron have focused on pathogens,^16,17^ whereas the gut microbiota is dominated by four bacterial phyla, Firmicutes, Bacteroidetes, Actinobacteria, and Proteobacteria, whose use of iron has been far less studied.^18^ Considering the huge individual variations in the composition of gut microbiota and the different iron requirements of gut bacteria, one would expect iron status and/or iron intake by the host to affect gut bacterial composition. In turn, any modification of the gut microbiota would have consequences for the health of the host. The aim of this review is to provide insights into the interplay between iron and gut microbiota in nonpathogenic conditions.

## Host iron metabolism

Humans are unable to actively excrete iron, so its concentration in the body has to be regulated at the iron absorption site in the proximal small intestine^1^. Absorption of dietary iron typically balances nonspecific losses, which occur via menstrual blood flows, and exfoliation of gastrointestinal epithelial cells. Adults absorb 25–50 g of dietary iron over their lifetime, which is an equivalent of about 1 mg of iron/day, this amount represents only about 10% of the iron present in food.^1^ Iron metabolism involves precisely regulated processes of absorption, cell use, recycling, and transport.

**Figure 1.**
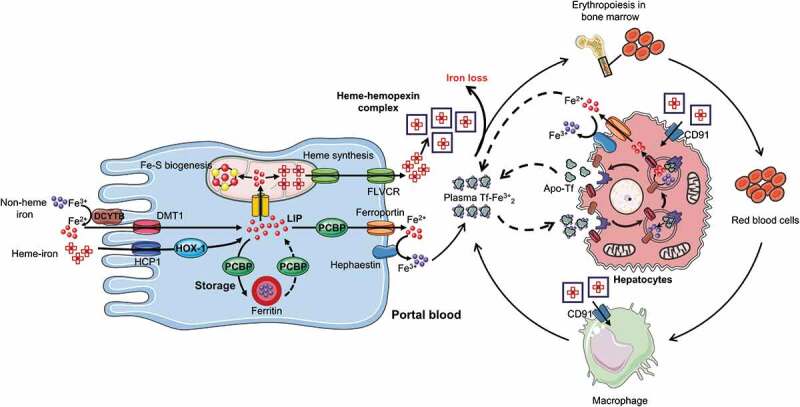
Enterocyte iron absorption and systemic iron distribution. Heme iron is transported in the enterocyte by Heme carrier protein 1 (HCP1), and Fe^2+^ is released in the cytoplasm by heme oxygenase 1 (HOX1) to join the labile iron pool (LIP). Fe^3+^ is reduced to Fe^2+^ by duodenal cytochrome b (DCYTB) in the brush border membrane of duodenal enterocytes, then Fe^2+^ joins the LIP via the divalent metal transporter (DMT1). Poly(rC)-binding protein (PCBP) delivers ferrous iron to the iron storage protein, ferritin, and vice versa. PCBP also transports Fe^2+^ from the LIP to the basolateral iron exporter, ferroportin. A small proportion of Fe^2+^ is also transferred to the mitochondria for synthesis of iron-sulfur clusters (Fe-S) and heme, which can be exported from the mitochondria and enterocytes using feline leukemia virus C receptor (FLVCR). The exported free heme is complexed with hemopexin into a heme-hemopexin complex, which can be directly absorbed by cluster of differentiation (CD91) receptors on the liver and macrophage. Exported Fe^2+^ is converted into Fe^3+^ by hephaestin, Fe^3+^ is then bound to transferrin to be transported to the bone marrow and hepatocytes for erythropoiesis and storage, respectively. This complex then binds to the transferrin receptor-1 (TfR1) on the cell surface of targeted cells and delivers its cargo to the cytosol via endocytosis. Apo-tf, transferrin without iron

As shown in the schematic diagram in Figure 1, in mammals, four intestinal iron transporters, the divalent metal transporter (DMT1), duodenal cytochrome b (DCYTB), ferroportin, and hephaestin are involved in absorption of non-heme iron.^19,20^ Exported iron is transported in the plasma linked to transferrin, and in healthy individuals, it is estimated that about 30% of transferrin is saturated with iron.^21,22^ The main regulator of systemic iron homeostasis is hepatic hepcidin, which prevents iron from entering the plasma from enterocytes and macrophages.^23^ In the following section, we give a more detailed description of iron absorption, use, transport, and homeostasis.

### Iron absorption, use, and transport

Iron absorption takes place in the duodenum and is regulated by two distinct pathways, one for heme and the other for non-heme iron. Heme bound iron is taken up at the border of the duodenal brush, but the exact mechanism remains unclear. Heme carrier protein 1 (HCP1) and the heme responsive gene (HRG1) have been suggested as potential uptake transporters for heme iron, but HCP1 was recently proposed to be a folate transporter unrelated to heme. However, a more recent study suggested that HCP1 is involved in low-affinity heme-iron uptake and not only in folate transport.^24^ In this case, heme oxygenase 1 (HOX1) activity would catabolize cytosolic heme into ferrous iron, which presumably then joins the cellular labile iron pool. However, the mechanism of transport of ferrous iron to the labile iron pool is not known. Dietary non-heme iron in its non-bioavailable oxidized (ferric) form must first be reduced to the ferrous form by DCYTB, after which DMT1, which is expressed in the brush border membrane of duodenal enterocytes, transports it across the intestinal epithelium.^25^

The iron present in the intestinal epithelial cell may either remain in the cell for use or storage (this iron is never absorbed into the body; rather, it is lost when enterocytes senesce and are soughed into the gut lumen) or exported across the basolateral membrane of the enterocyte into circulatory system (absorbed iron).^26^

In the enterocytes, iron chaperone poly(rC)-binding protein 1 is responsible for delivering ferrous iron to ferritin.^27^ Ferritin is a spherical heteropolymeric protein composed of 24 subunits.^28^ Iron may leave ferritin through gated pores or via autophagy and lysosomal degradation of ferritin.^29,30^ Iron storage in ferritin is largely determined by the level of ferritin protein, which is tightly regulated by cellular iron levels.^31^ The ferritin compartment that stores iron in enterocytes may be required for controlled delivery of iron to the basolateral iron exporters,^32^ but other systems may exist in addition to this well-described absorption mechanism. For example, a small amount of ferrous iron may be transferred directly from endosomes to mitochondria, while the remainder enters a metabolically active pool in the cytosol, termed labile iron pool, that ends in the mitochondria.^26,31^ In the mitochondria, iron is incorporated in heme or produces iron-sulfur clusters (Fe-S) that bind with mitochondrial enzymes. However, heme and some products of the Fe-S clusters assembly machinery are also exported from the mitochondria for use in the cytosol, nucleus, and other membrane-bound organelles. Cytosolic iron that is not directed to mitochondria may be used to metallate non-heme iron enzymes of the cytosol and nucleus, to assemble cytosolic Fe-S clusters, or be stored and sequestered in ferritin.^31^

Iron is exported from the cell by a major protein, ferroportin, that is present on the basolateral membrane.^25,33^ Ferroportin is mainly expressed by cells and tissues associated with iron transfer to plasm, i.e., duodenal enterocytes, liver Kupffer cells, splenic red pulp macrophages, periportal hepatocytes, and the placental syncytiotrophoblast.^34^ Following export of ferrous iron across the basal membrane by ferroportin, it is then oxidized by a multi-copper oxidase protein called hephaestin before being bound by plasma transferrin.^35^ Other proteins such as breast cancer resistance protein (Bcrp)/ATP-binding cassette subfamily G member 2 (Abcg2) and feline leukemia virus C receptor (FLVCR) have been reported to be responsible for the export of heme iron out of the cell, but although, but some heme iron traverses the cells intact, the underlying mechanism is still not known.^36^ The exported free heme is complexed with hemopexin into a heme-hemopexin complex, which can be directly absorbed by cluster of differentiation (CD91) receptors on the liver and macrophage.^37^ Iron released into the circulatory system binds to transferrin in the blood, which then binds to transferrin receptor-1 (TfR1) on the cell surface and delivers its cargo to the cytosol via endocytosis.^22^ In these more acidic conditions, iron dissociates from transferrin and is then reduced by ferrireductases to cross the endosomal membrane via DMT1. Another transferrin receptor, the type 2 transferrin receptor (TfR-2), largely restricted to liver and erythroid precursors, also interacts with holo-transferrin but does not substantially contribute to iron uptake, rather as a sensor of systemic iron status.^38,39^

Iron is also absorbed in the ileum and colon; the expression of specific proteins involved in iron absorption, including ferroportin, DMT 1, and ferritin has already been described, and the subsequent transferability of the iron to the venous blood is discussed in Ref. 40. However, the relative contribution of these sites to overall iron absorption is probably small depending on the site and on the iron status of the individual.^41^

### Iron homeostasis

Iron homeostasis in mammals is mainly regulated by a set of interlocking regulatory systems that include hepcidin-ferroportin mediated regulation of serum iron levels, control of intracellular iron levels by transcriptional regulation (hypoxia-inducible factors (HIF), and iron regulatory proteins (IRPs)).^42^

**Figure 2.**
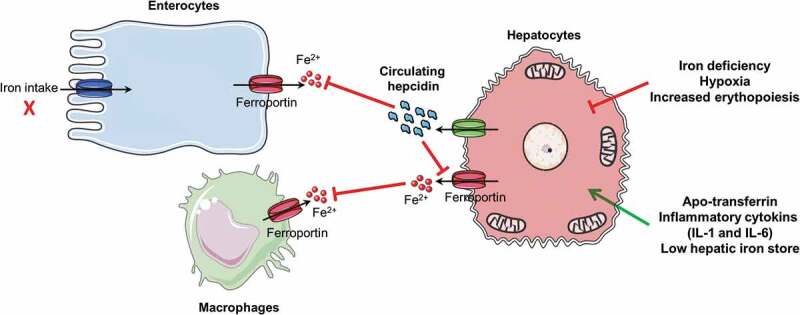
Essential model of the role of hepcidin in maintaining iron homeostasis. Hepcidin produced in the liver downregulates ferroportin expression in enterocytes, macrophages, and hepatocytes in case of inflammation, low iron store, and low transferrin level. The red arrow signal inhibition of hepcidin and the green arrow signal expression of hepcidin. IL 1 and 6, interleukin 1 and 6

Hepcidin, a 25 amino acid peptide hormone secreted by hepatocytes that circulates in the plasma plays a crucial role in maintaining systemic level iron homeostasis.^43^ Hepcidin inhibits the release of iron into the plasma from three main sources: dietary absorption in the duodenum, the release of recycled iron from macrophages, and the release of stored iron from hepatocytes (Figure 2).^23^ Hepcidin inhibits the release of iron into the circulatory system by post-translationally regulating its cognate receptor ferroportin. Hepcidin is regulated at transcriptional level by multiple signals including systemic iron levels, stores of hepatic iron, erythropoiesis, hypoxia, and inflammatory/infectious states.^44^ More precisely, two pathways, manly involving human homeostatic iron regulator protein (HFE) and bone morphogenetic protein (BMP), mediate hepcidin regulation via systemic iron levels and intracellular iron stores. High systemic iron concentrations result in displacement of HFE from TfR1 to TfR2, resulting in up-regulation of hepcidin expression.^45^ BMP controls iron stores in hepatocytes; BMP is repressed by iron deficiency and activated by increased iron levels. This signaling pathway is initiated upon BMP binding to a BMP receptor complex on the cell surface, which activates the receptor kinase to phosphorylate different cytoplasmic proteins that are homologs both of the *Drosophila* protein mothers against decapentaplegic (MAD) and of the SMA protein of *C. elegans* (SMAD1, SMAD5, and SMAD8). SMAD1, 5, and 8 then form transcription factor complexes with SMAD4 that translocate to the nucleus and trigger the transcription of different target genes including HAMP (hepcidin antimicrobial peptide). Hepatic hemojuvelin protein (HJV), or hemochromatosis type 2 protein (HFE2), is an indispensable BMP co-receptor and induces hepcidin expression via the BMP signaling pathway.^45^

Erythropoiesis requires considerable quantities of iron, and the inhibition of hepcidin expression by erythropoietic signals is thus of considerable physiological significance. For example, hepcidin suppression in response to phlebotomy or hemolysis has been shown to depend on intact erythropoietic activity in a mouse model, where irradiation and cytotoxic inhibition of erythropoiesis prevented hepcidin suppression. However, the molecular mechanisms and the other factors involved are still poorly understood.

The inflammatory cytokines interleukin-1 (IL1) and interleukin-6 (IL-6) are both potent inducers of hepcidin expression. IL6 activates the Janus kinase (JAK)/signal transducer and activator of transcription (STAT) signaling pathway, which in turn, activates the hepcidin promoter via a STAT-binding motif close to the transcription start site. The BMP signaling pathway also contributes to the inflammatory response via SMAD4.^23^

Very recently, conventional dendritic cells (cDCs) were found to produce hepcidin in inflamed intestine of mice induced by microbiota-derived signals and to subsequently to limit iron release from intestinal phagocytes to prevent tissue infiltration by the microbiota, and hence to promote mucosal healing. However, the impact of hepcidin produced by cDCs on systemic iron homeostasis and immune responses has not yet been established.^46^

Independently of iron stores in the body, tissue hypoxia also inhibits hepcidin expression in hepatocytes by inducing erythropoiesis. The central mediators of hypoxia-induced erythropoiesis are HIF proteins, among which HIF-1α and HIF-2α are the best characterized. HIF-1α binds to the enhancer element of the erythropoietin (EPO) coding gene and activates its transcription in response to hypoxia. EPO synthesis in turn stimulates erythropoiesis.^47^

Regulation of cellular iron homeostasis is also controlled at the level of the transcription of genes encoding proteins involved in iron metabolism. One of the main regulators of these changes in transcription is HIF2α.^48^ HIF-2α is an oxygen- and iron-regulated transcriptional factor that directly targets the three key intestinal iron transporters DMT1, DCYTB, and ferroportin, by increasing their transcription.^49^

IRP1 and IRP2, both mRNA binding proteins,^50^ coordinate the regulation of cellular iron uptake, storage, efflux, and erythroid use by cells. They interact with iron-responsive elements (IREs) that are present in the 5ʹ and 3ʹ untranslated regions of the target mRNAs.^51^ In iron-starved cells, IRP1 and IRP2 bind to the IRE in ferritin mRNA, thus preventing their translation, to the TfR-1 mRNA to protect it against endonucleolytic degradation. This increases cellular iron uptake from transferrin and prevents storage of the metal. By contrast, in iron replete cells, the IRE-binding activities of IRP1 and IRP2 are reduced, enabling TfR-1 mRNA degradation and translation of ferritin mRNA. This further inhibits iron uptake and stimulates storage of excessive intracellular iron within ferritin.^51^

### Major pathophysiologies associated with the dysfunctional iron homeostasis

The most studied pathophysiologies were iron overload and iron deficiency.^53^ Iron overload occurs in two diseases, hereditary hemochromatosis and hemoglobinopathy-related anemia, which is also called iron-loading anemia.^52^ When mutation occurs in gene coding hepcidin itself or in genes that are major inducers of hepcidin activating pathways (TfR-2 and HFE2), low levels of hepcidin and systemic iron overload (hemochromatosis) are observed.^53^

Iron deficiency mainly results from an unbalanced iron uptake and loss; increasing dietary iron intake is efficient in many cases. Nevertheless, expression of hepcidin can occur due to different pathologies, thus causing a reduction of iron absorption in the intestine.^52^ Causes can be genetics, with specific mutation on protease (transmembrane protease serine 6); or due to chronic inflammation due to cancer or inflammatory diseases. In the last case, activation of IL6, SAT, and SMAD induces hepcidin expression.^52^

## Bacterial iron metabolism

Iron metabolism in bacteria is detailed in a number of articles and has also been reviewed several times.^54–56^ Here we summarize the most frequently described processes.

Iron is generally found in one of the two redox states: oxidized (ferric form) or reduced (ferrous form).^57^ Under aerobic conditions and at the physiological pH of 7, ferrous iron is spontaneously oxidized into ferric form, which is hexacoordinated and forms soluble complexes with water molecules. These complexes can be hydrolyzed and produce polymeric iron hydroxides that eventually precipitate. Ferrous and ferric iron are in equilibrium.^57^

**Figure 3.**
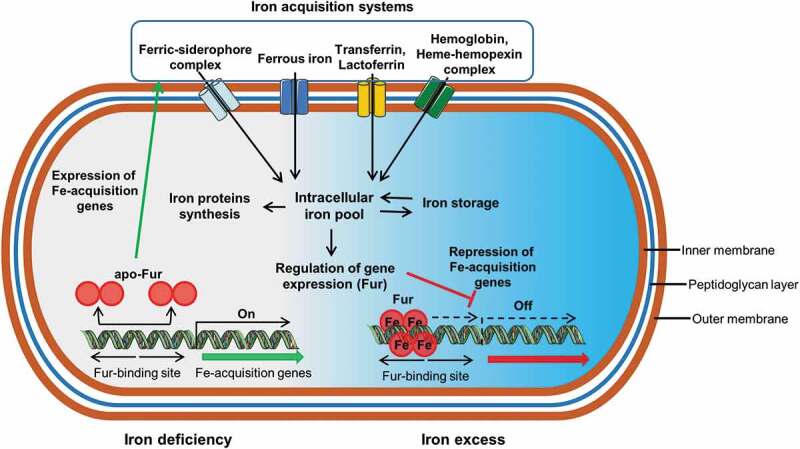
General iron uptake system and ferric uptake regulator (fur)-mediated iron uptake regulation in bacteria. Bacteria can acquire iron ferric iron complexes with siderophores, ferrous iron, transferrin, lactoferrin, hemoglobin and heme complexes using different receptors. Once inside the cell iron is stored, used for protein synthesis and for regulation of the expression of the gene Fur. In presence of iron, Fur forms a complex with Fe^3+^, which binds to the Fur biding sites of bacterial DNA to repress transcription of the genes involved in iron transport. In absence of iron, Fur cancels out repression and the genes are expressed

Bacteria use three major strategies to acquire iron: by producing and using siderophores (ferric-specific chelators), by absorbing ferrous iron after reducing ferric iron if necessary, and by using host iron compounds such as heme and transferrin (Figure 3).

### Bacterial iron absorption through siderophores

**Figure 4.**
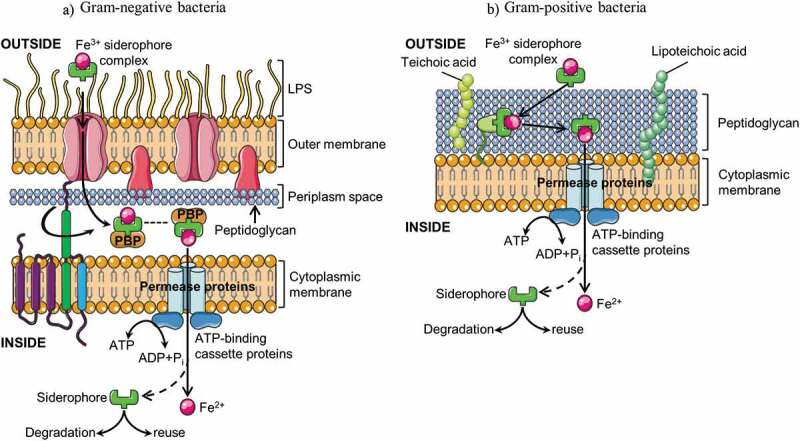
Schematic representation of bacterial siderophore-mediated iron uptake in Gram-negative (a) and Gram-positive bacteria (b). Ferric-siderophore complexes are internalized via specific outer membrane receptors, a periplasmic binding protein (PBP), and inner membrane ATP-binding cassette (ABC) transporters. In gram-positive bacteria, the energy required for iron uptake is satisfied by the coupling the proton motive force of the cytoplasmic membrane to the outer membrane via the TonB system (TonB, ExbB, ExbD). LPS, lipopolysaccharides

Siderophores are small, ferric iron-chelating molecules produced and secreted by many microorganisms in response to iron limitation caused by environmental iron deficiency.^58^ Hundreds of siderophores had been characterized and can be classified in four groups based on their function: catecholate, hydroxamate, phenolate, and carboxylate.^59^ Siderophores produce a ferric-siderophore complex that is subsequently internalized in the cell of the bacterium (Figure 4).

In Gram-negative bacteria, ferric-siderophore complexes are internalized via specific outer membrane (OM) receptors, a periplasmic binding protein (PBP), and an inner membrane ATP-binding cassette (ABC) transporter (Figure 4a). OM siderophore receptors are induced by iron deficiency and are consequently not present in iron-sufficient conditions. The ligand-binding sites of the receptors are specific to each siderophore. However, bacteria have multiple OM receptors, thus enabling the use of siderophores, which they are unable to synthesize themselves. Gram-negative outer membrane lacks an established ion gradient or ATP to provide the energy for transport. This energy requirement is satisfied by coupling the proton motive force of the cytoplasmic membrane to the outer membrane via three proteins, TonB, ExbB, and ExbD.^58^ Periplasmic binding proteins shuttle ferric-siderophores from the OM receptors to CM ATP-binding cassette (ABC) transporters, which in turn, deliver the ferric-siderophores to the cytosol where the complexes are probably dissociated by reduction.^54^

Conversely, Gram-positive bacteria lack an outer membrane, a cell wall composed of murein, polysaccharides, teichoic acids, and only cell wall proteins separate the bacterial cytoplasm from its environment (Figure 4b). Iron is uptaken by membrane-anchored binding proteins that direct the iron to a permease and ABC transporter system.

### Bacterial ferrous iron absorption

Bacteria are also able to transport ferrous iron, which is the most abundant form of iron in anaerobic conditions, or at low pH. Ferric iron can also be reduced to the ferrous form by extracellular reductases and is then transported by entirely different routes than those used for ferric iron.^60^

Feo is the widely distributed dedicated ferrous iron transport system in bacteria.^61^ The feo system was first identified in *E. coli* K12 and was found to be coded by the *feoABC* gene.^62–64^ FeoB is an integral IM protein whereas FeoA and FeoC are probably cytoplasmic. Feo systems play an important role in bacterial virulence in environments where the oxygen concentration is low. This was demonstrated by deleting the *feo* genes in many pathogenic/nonpathogenic Gram-negative and Gram-positive bacteria. For instance, when *feo* genes are deleted, strains of *Escherichia coli, Helicobacter pylori*, and *Campylobacter jeuni* were shown to be unable to take up ferrous iron or to colonize the mouse intestine. On the contrary, it has been shown that other pathogenic bacteria such as *Escherichia coli* 08 strain x7122, and *Shigella flexneri* survived regardless of the deletion of this gene, strongly suggesting that another mode of iron transport is at play here.^62^

Other transporters of ferrous iron including zinc-regulated transporters (ZIP-like transporters), natural resistance-associated macrophage protein (Nramp) transporters, EfeUOB systems, and P19 iron transporters are also described.^54^

Bacteria also have receptors for host transferrin and lactoferrin, and can consequently absorb them directly (Figure 3). These receptors are located in the outer membrane and are induced by iron starvation. In this case, iron is stripped from transferrin and lactoferrin at the bacterial cell surface and the iron-free proteins are released extracellularly. The transport of the iron released from transferrin and lactoferrin across the periplasm and cytosolic membrane depends on a periplasmic binding protein ABC permease system.^54^

Some pathogenic bacteria also have heme acquisition systems; heme is liberated from red blood cells by hemolysins and protease. Upon release, heme may be bound by host proteins (hemopexin, albumin); however, it can also be directly absorbed by bacteria.^54^ Extracellular iron is not the only source of iron in bacteria since they can also make use of intracellular iron, especially in iron-restricted conditions. Three types of iron storage proteins have been identified in bacteria, ferritin, heme-containing bacterioferritins, and the smaller iron detoxification proteins that protect the chromosome from iron-induced damage by free radicals.^54^

### Other mechanisms

A few organisms were thought to not require iron. For instance, lactic acid bacteria were generally considered not to require iron since no difference in growth was observed in different *Lactobacillus* strains grown in iron repleted and depleted media.^65,66^ In addition, *Lactobacillus plantarum* was able to grow in iron-restricted media obtained by removing 95% of iron and adding ethylenediamine di-*O*-hydroxyphenyl acetic acid (EDDA). The same study also showed that cells of *Lactobacillus plantarum* contained less than 0.1 µm intracellular iron, far less than *Escherichia coli*, which have high-affinity Fe acquisition systems.^67^

Conversely, some studies showed that *Lactobacillus sakei* (nonpathogenic bacteria found in meat) have complete genetic equipment dedicated to the transport and use of iron. A microscopy approach revealed that this species could use iron sources present in its natural ecosystem including myoglobin, hemoglobin, hematin, and transferrin, to ensure its long-term survival during stationary phases. Still, the presence or absence of iron did not affect growth, showing that iron is not indispensable for growth, but that the presence of the above-mentioned iron sources enhanced its survival during stationary phases.^68^ Some putative metal iron ABC transporters and energy coupling factors (ECF) identified as responsible for transport, constitute a novel family of conserved membrane transporters in prokaryotes whose domain organization is similar to that of ATP-binding cassette transporters. This is the first description of the involvement of ECF in the heme transport system.^69^ The metabolism of iron is usually studied in pathogenic species, as it is considered as a virulence-associated factor in the infected hosts, whereas information concerning nonpathogenic bacteria is scarce, and so studying them may reveal new pathways.

### Regulation of bacterial iron uptake

Bacteria generally regulate their iron metabolism in response to iron availability, this regulation being mediated by the ferric-uptake regulator protein (Fur) that controls the iron-dependent expression of genes (Figure 3).^70^ In presence of iron, Fur forms a complex with ferric iron and the complex binds to the Fur boxes of bacterial DNA to repress transcription of the genes coding for proteins involved in iron transport. In absence of iron, Fur cancels out repression and the genes are expressed.^54^

Nevertheless, the repertoire of gene regulatory function is more expanded. Indeed, Fur can also act as a positive regulator of gene transcription, through repression of regulatory RNA or activation of gene expression which will prevent the recruitment of repressors. It can also directly activate gene expression. Fur can also act as a transcription activator and repressor in the absence of iron, but this was observed in a limited number of pathogenic bacteria.^71^ Fur also regulates bacterial iron storage.^54^ On the whole, Fur is more than a simple repressor of iron uptake since it integrates several biological pathways (e.g. expression of virulence factors, survival mechanisms to resist acid and oxidative stresses), contributing to the virulence of bacterial pathogens.^71^

## Role of iron in host-microbe interactions

The intestinal microbiota is composed of trillions of organisms belonging to hundreds of different species. It consists of bacteria, archaea (single-celled organisms with no nucleus that are more closely related to eukaryotes than to bacteria), fungi (mostly yeasts), microbial eukaryotes, and viruses/phages.^72^ In humans, bacteria dominate the gut microbiome. Five bacterial phyla (Firmicutes, Bacteroidetes, Actinobacteria, Proteobacteria, and Verrucomicrobia) represent the majority of bacteria that comprise the gut microbiota.^18^ Less prevalent phyla are Cyanobacteria, Fusobacteria, Lentisphaerae, Spirochetes, and TM7.^18^

The bacteria in the gut provide functional traits that humans have not evolved on their own.^73^ Several metabolic, physiological, and immunological features depend on mutualistic associations with the intestinal microbial community. As the microbiome encodes more digestive enzymes than do its hosts, it helps the host by breaking down indigestible macromolecules (polysaccharides, etc.) or by synthesizing certain vitamins. It is also involved in the development of the immune system, maturation of epithelium cells, and in protecting the host against pathogens by providing resistance to colonization.^74–76^ Among the factors that affect the bacterial composition in the gut is the presence and the extent of available substrate, or the absence of substrate in the environment. As iron is a growth-limiting factor, it is crucial for the growth and proliferation of most bacteria and has a direct impact on host–microbiota interactions.

In mammals, the lower gastrointestinal tract, i.e. the cecum and colon, contains a variety of distinct microbial habitats with different microbial densities. First, microbial density increases from the proximal to the distal gut, the stomach contains 10^1^ bacteria/g of content, the duodenum 10^3^ bacteria/g, the jejunum 10^4^ bacteria/g, the ileum 10^7^ bacteria/g, and the colon up to 10^12^ bacteria/g. The microbial community in the small intestine is dominated by fast-growing facultative anaerobes that tolerate the combined effects of bile acid and antimicrobial compounds produced by the host. The microbiota in the small intestine plays different roles for the host although its role in iron metabolism is not yet established. In general, it is less studied than microbiota in the colon, partly due to its inaccessibility and the invasiveness required to study it.

Despite its importance in numerous cellular processes, free iron can be toxic at high concentrations due to its redox potential. Consequently, virtually all organisms tightly regulate the uptake and storage of iron.^77^ In humans, the amount of free iron is extremely low (~10^−24^ M) in extracellular microenvironments such as blood vessels, interstitial spaces, or epithelial surfaces. The concentration of free iron is assumed to be at least 8-fold lower than that required for microbial growth.^78^ Therefore, most of the iron is bound to high-affinity host proteins such as ferritin, haptoglobin, hemopexin, lactoferrin, transferrin, and lipocalin, which can make iron unavailable for microbes.^79^

However, high concentrations are expected in the human colon given that the majority of iron is not absorbed and will end up there. This has been confirmed in a few studies. For instance, fecal iron excretion in adults who consumed a diet containing a normal concentration of iron (6–8 mg/day) was about 7.5 mg/day (1.3 × 10^–4^ M/day). Fecal iron excretion was higher, about 15 mg/day (2.7 × 10^–4^ M/day), when the iron intake was higher, 11–15 mg/day.^80^ Another study on weaning infants on a standard Western diet reported a fecal iron concentration of about 1.6 × 10^–4^ M and fortified iron 4.8 × 10^–4^ M.^81^ Considering that bacteria require a minimum of ~10^−6^ M to grow, these studies show that the concentration of iron in the colon is higher than the amount required for bacterial growth. However, the bioavailability of luminal iron for bacteria not only depends on total iron content, but on a range of different factors including the form of iron (heme and non-heme), iron speciation (oxidation, mineralization, and the presence of metal-binding ligands), pH, and oxygen levels.^82^ Heme-iron in hemoglobin and myoglobin is more bioavailable than non-heme iron. Non-heme iron is the most affected by the composition of the food matrix and the physical-chemical conditions of the intestinal lumen.^83^ Therefore, the availability of iron for gut microbiota in the colon is extremely difficult to predict and has been less studied due to the difficulty involved in measuring it.^82^ In general, the amount of iron available for microbes is calculated as the sum of external and host-derived sources.

One study investigated iron speciation in the feces after iron supplementation.^84^ Of the ~3 × 10^–4^ M/100 g of iron found in the feces, about 30% was potentially bioaccessible. Apart from the residual non-bioaccessible fraction (74.8%), bioaccessible species of iron were distributed as follows: carbonate-bound (acid-soluble, 20%), oxide-bound (reducible iron, 6.89%), organic (oxidizable iron, non-detectable) and exchangeable (2.75%) fraction.^84^

Several dietary factors can influence the bioaccessibility of iron for the host and bacteria. For example, ingestion of non-digestible carbohydrates such as fructo-oligosaccharides results in the production of short-chain fatty acids (acetate, propionate, and butyrate). These can lower the luminal pH, in turn leading to the reduction of iron to the soluble ferrous form or even altering ligand composition (carbonate bound to exchangeable and oxide fractions).^84,85^

Inhibitors, such as phytates, polyphenols, and tannins, can also reduce iron bioaccessibility. Many bacteria can also indirectly improve iron bioaccessibility by enzymatically degrading chelators such as tannins or phytates.^83^ On the contrary, promoters such as ascorbic acid from the meal or organic acids synthesized by bacteria (lactic and propionic acids, etc.) can improve iron bioaccesibility. Therefore, the whole meal has an effect on iron bioaccessibility.

The interplay between the host and the bacteria is dynamic since many actions can happen at the same time and the outcome is difficult to predict. The host can also modify iron uptake by bacteria by synthesizing lipocalin, which will lead to absorption of siderophores and hence imitate iron absorption by bacteria. This has been extensively studied in cases of inflammation, but it is possible that it also occurs in the normal state. Some authors suggest that commensal bacteria provide iron (that would otherwise not be bioavailable) to the human host via these mechanisms. Furthermore, with all their equipment to capture or liberate iron, it is possible that commensal bacteria share iron among themselves as well as with the human host.^86–88^ Mutual understanding between host and bacteria may result in mutual adaptive changes to ensure the maintenance of appropriate levels of iron over the lifetime of the organism. Both the host iron status and dietary iron availability affect this microbial ecosystem.^6,7,89^ As the main strategy to fight iron deficiency is iron supplementation, this will also affect the gut microbial compartment.

## Effect of iron supplementation on gut bacterial composition

**Figure 5.**
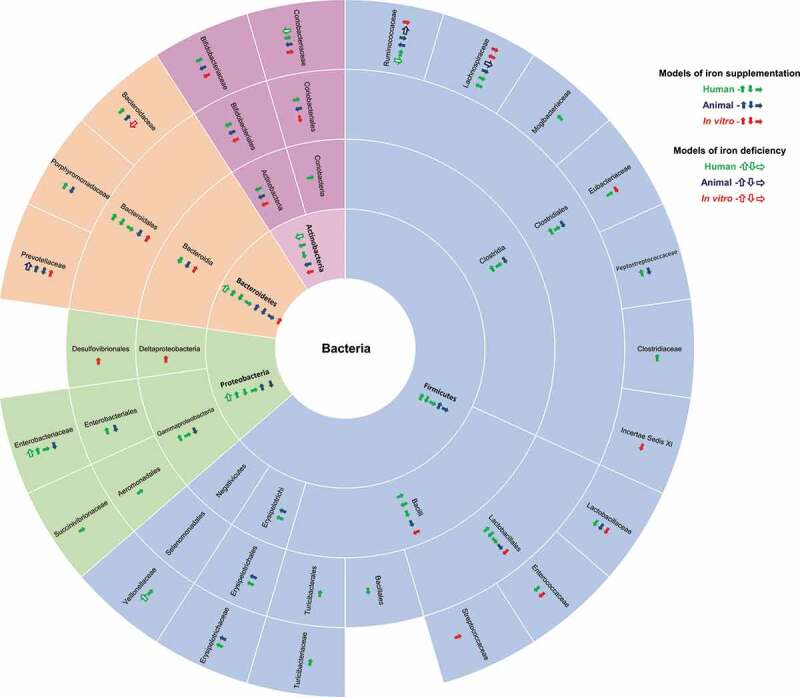
Summary of the effect of iron supplementation (plain arrows) and iron deficiency (empty arrows) on fecal bacterial composition at different taxonomic levels. Results are from human (green arrows), animal (blue arrows) and in vitro (red arrows) experiments. The direction of the arrow indicates the effect: the arrow up indicates an increase in the proportion of the taxon, the arrow down indicates a decrease of the proportion of the taxon, and the arrow to the right indicates an absence of effect on the proportion of the taxon.^6,9,10,89–108^

The effect of iron supplementation on the composition of gut microbiota has been the subject of around 20 different articles. In most cases, high throughput sequencing of the 16S rRNA coding gene was used to investigate the microbiota and the results of the analyses of this literature are presented in Figure 5. The results of detailed analyses of the data available on the four most abundant phyla (Firmicutes, Bacteroidetes, Actinobacteria, and Proteobacteria) among gut bacteria are presented according to the phylogenetic tree structure. Additional information obtained using different methods is given in the text.

The four phyla have not been studied to the same extent since the effect of iron supplementation on Firmicutes is the most documented, followed by the effects on the phyla Bacteroidetes, Proteobacteria, and Bifidobacteria using *in vitro*, animal and human models. Only a few articles have been published on each model. For example, among the eight articles reporting the effect of iron supplementation on the Firmicutes phylum, six were on humans and two on mice (Figure 5), ^10,89–93,108^ What is more, adding iron using different models had either no effect, or increased or reduced the proportion of the different phyla, making generalization difficult (Figure 5). A detailed analysis of one model also revealed inconsistencies. For example, in humans, an increase,^10^ a decrease^91^, and no effect^89,91,109^ of iron administration were reported for the phylum Firmicutes (Figure 5). These discrepancies appeared at all taxonomic levels. For example, at the **order** level in humans, the proportions of Lactobacillales or Bacteroidales were reported to increase,^10^ decrease^94^, or to remain unchanged^91^ (Figure 5). Iron supplementation was reported to modify or to have no effect on the proportion of different families, genera, and species depending on the model used in the study and variations occurred in different ways. The proportions of some genera in the Firmicutes phylum were systematically reported to remain unchanged: *Bacillus, Turicibacter, Granulicatella, Megamonas, Veillonella, Turicibacter, Candidatus Arthromitus* (SFB), *Butyrivibrio, Subdoligranulum* and *Clostridium cluster IV*;^95,96^*Alistipes*^95^ in the Bacteroidetes phylum; *Enterobacter, Succibivibrio, Desulfovibrio*, and *Parasutterella*^91,92,95^ in the Proteobacteria phylum; *Colinsella, Eggerthella*, and *Rothia*^93,97^ in the Actinobacteria phylum. The proportion of some genera was reported to increase in all studies, including *Dialister, Lachnospira, Clostridium cluster XIVa, Oscillibacter, Xylanibacter, Helicobacter, Shigella*, and *Slackia*;^91,95,96,98,99^ while the proportion of other genera was reported to decrease in all studies, including *Pediococcus, Phascolartobacterium, Sporacetigenium, Oscillospira, Acetanaerobacterium, Barnesiella, Asaccharobacter*, and *Alloscardovia*.^93,96,89^ The results of the analyses of other genera were inconsistent.

One possible explanation for the consistent results may be the limited amount of data available, as in the case of the decrease in the proportion of the *Barnesiella* genus, which was the subject of only one study.^93^ In contrast, in frequently studied genera such as *Bacteroides*, the results reported were highly variable. Indeed, in humans and in mice, iron supplementation has been reported to have no effect,^98^ to increase,^10,89^ or to reduce the proportion of the *Bacteroides* genus.^95,106^ The same goes for the widely studied genus *Lactobacillus*, whose proportion was reported to remain stable, increase, or decrease depending on the study.^6,93,96–98,106^

### The form of iron affects fecal bacterial composition

A range of methods is used to combat anemia in large-scale programs in low- and middle income countries. Ferrous sulfate, ferrous fumarate, and elemental iron are most frequently used for fortification and have different characteristics that are taken into account in their use. Ferrous sulfate and ferrous fumarate are characterized by high bioavailability, although they may turn rancid or change in flavor or in color. Although only half as bioavailable, ferrous sulfate does not undergo physical and sensory changes.^109^ The chelated iron complex, ferric sodium ethylenediaminetetraacetate (NaFeEDTA) is frequently used since it is highly bioavailable due to its inert chemical reactivity to lipid peroxidation and resistance to luminal inhibitors such as phytate.^109,110^ Nano iron has also been tested as it does not require solubilization in the stomach prior to uptake by enterocytes as whole nanoparticles via endocytosis.^111^

The different levels of bioavailability of the different forms of iron used in supplementation and fortification lead to different modulation of the gut microbiota. For instance, one study showed that dietary repletion in rats fed with ferrous sulfate had more impact on the gut microbiota than electrolytic iron.^100^ Similarly, iron supplementation using hemin *in vitro* led to different microbiota profiles than ferrous sulfate and citrate.^96^ Furthermore, iron supplementation using similar concentrations of ferrous sulfate, ferrous bisglycinate, and NaFeEDTA showed distinct clustering of microbiota communities in a model of colitis in mice.^112^

Conversely, fecal microbiota in rats supplemented with Nano Fe III appeared to have similar bacterial diversity to that in rats supplemented with ferrous sulfate.^95^ Iron supplementation using ferrous sulfate or ferrous citrate also resulted in a similar microbiota profile *in vitro*.^96^ On the whole, the results of the effects of different iron formulations on gut microbiota composition appear to be contradictory, probably due to the limited number of studies available, further studies are therefore required.

### Iron deficiency and bacterial gut microbiota

The effects of iron deficiencies on the composition of bacterial gut microbiota are summarized in Figure 5. In the case of iron deficiency, it would be logical to expect the opposite effect from that of iron supplementation. However, based on the highly variable results reported for iron supplementation, iron deficiency also modifies the gut bacterial composition in contradictory ways. In some cases, the results appear to be logical, for example, the proportions of *Dialister* and *Helicobacter* genera were reported to increase with iron supplementation and to decrease in the case of iron deficiencies in different models.^91,95^ However, this is not always the case, since, for example, a decrease in the proportion of the *Lachnospiraceae* family due to both iron supplementation^93,95^ and iron deficiency were reported using a mouse model.^95^ In two human studies, iron supplementation did not affect the proportion of *Veillonellaceae* family,^91^ whereas iron deficiency increased it^101^ (Figure 5). Again, the genus *Bacteroides* was one of the most frequently studied genera, and results differed even when the same models were used.^95,99^ One interesting study also mimicked extreme iron deficiency *in vitro* using chelators and reported a decrease in the proportion of the *Bacteroidaceae* family.^99^

Again, no general trend could be identified, even in the same model, at a given taxonomic level, and using the same methods. However, it is important to note that even in one model, certain differences may partly explain the different effects of the host iron status on the composition of the gut microbiota. For example, in studies on humans, different populations were targeted (neonates, infants, and adults).^91,98, 101,106^ Different rodents were also used (mice, rats) and among rats, for example, different genetic backgrounds were used (Wistar, Sprague-Dawley rats, and BalbC and C57BL/6J mice).^93,95,100^ As was the case with iron supplementation, the apparently consistent results may only be due to the limited amount of data available.

### The case of the Lactobacillaceae family and the Actinobacteria phylum

We were able to find some rare consistent results. One example being that the proportion of *Lactobacillaceae* family always decreased during iron supplementation, whatever the model used (Figure 5).^10,93,96^ The other example concerns the phylum Actinobacteria, which was the subject of eight studies (Figure 5), of which five were on humans, two on mice, and one used an *in vitro* model. In humans, one of the five studies reported a decrease in the proportion of the Actinobacteria phylum in the case of iron-deficiency anemia.,^101^ while the other four studies focused on the effect of iron supplementation. Three of the studies on human infants and toddlers reported no effect of supplementation on the Actinobacteria phylum.^92,109,113^ On the contrary, a decrease was observed during iron supplementation in infants,^10^ in mice,^93^ and in the *in-vitro* model.^96^ All three studies that dealt with the effect of iron supplementation on the Actinobacteria class, Actinobacteriales order, and *Bifidobacteriaceae* family described a decrease in the proportions, regardless of the model used.^10,93,96^ At the genus level, results were less consistent, since depending on the study, the administration of iron did not influence or reduced the proportion of *Bifidobacterium* regardless of the method used to detect the bacteria (culture, real-time PCR, PCR-TGGE, and 16S rRNA sequencing).^9, 10, 90, 91, 93, 95, 96, 89, 106, 114^

A better understanding of iron requirements and use by bacteria should help explain this consistency.

### Microbiota modulates iron metabolism

In the previous sections, we described modulation of gut microbiota by iron supplementation and deficiency. In addition, gut microbiota also modifies host iron absorption and homeostasis. For instance, germ-free rabbits and mice had less iron stored in their liver, spleen, and kidney than their conventional counterparts.^103,115^ Additionally, germ-free mice showed a decrease in iron absorption that was calculated in two ways, one by calculating the difference between ingested and excreted iron,^104^ the other by calculating the increase in iron absorption expressed at mRNA and protein levels (Dcytb and Dmt1), and the decrease in the iron storage protein (ferritin)^115,116^ and in basolateral export proteins (ferroportin) in the duodenum and colon.^116^ This supports the hypothesis that commensal bacteria in the gut liberate a pool of iron from which the human host is capable of drawing benefits.

The addition of a microbiota to the initially germ-free animals was also shown to have an effect on the host’s iron metabolism. Indeed, conventionalization of germ-free rats increased body iron retention by 25%^104^ and decreased Dcytb and Dmt1 mRNA expression.^115^ A decrease in Dcytb, Dmt1 and hephaestin in protein upon conventionalization has also been reported elsewhere.^116^ Further, the pattern obtained after mono-colonization in different species (*Bacteroides thetaiotamicron, Faecalibacterium prausnitzii*, and the probiotics *Streptococcus thermophilus*) used individually was similar to the pattern observed in the conventional mice experiment. This led to the conclusion that the response was generic since it was not specific to a single bacterium.^116^

Administration of iron to iron-deficient germ-free animals improved their iron status in the same way as in conventional animals. When iron-deficient germ-free rabbits received a diet containing a larger proportion of iron citrate or iron than they can obtain from natural sources (soymeal) their iron status improved, as shown by an increase in hemoglobin and hematocrit.^103^ Intravenous administration of iron in germ-free and conventional mice also resulted in similar plasma iron levels.^105^

A very recent study used a very complete combination of germ-free and conventional mice fed iron-sufficient or iron-deficient diets.^116^ The authors showed that gut microbiota competed with the host for iron, particularly in iron-limited conditions. They concluded that gut microbiota regulates systemic iron homeostasis by repressing the intestinal iron absorption pathway and by promoting cellular iron storage (ferritin) through the production of specific bacterial metabolites. These metabolites were identified as 1,3-diaminopropane and reuterin and inhibited HIF-2α activity through inhibition of heterodimerization, thus reducing systemic iron overload.^19,115^ The administration of a bacterium able to synthesize such compounds (reuterin) is an efficient way to modulate iron metabolism in mice with inducible iron metabolism disorder related to hepcidin.^115^ Elucidating the benefits of individual microbiota-derived molecules in host animals is important for understanding the symbiosis between humans and their microbiota.

## Conclusion

It is clear from our literature review that iron/gut microbiota/host interacts intimately. Profound changes in the composition of gut microbiota have been reported depending on iron status but the patterns of change varied with the study, the model used, and/or the form of iron. For other cross-talk between bacteria and the host, the initial composition of the microbiota has been shown to be the most important factor involved, and this is perhaps also true for iron.^117^ Almost all studies on iron uptake by bacteria have focused on pathogenic bacteria. Nevertheless, some mechanisms may be shared with commensal bacteria and the question remains as to how the host differentiates between commensal and pathogenic bacteria. The most recently published study showed that it is possible to use microbiome-based therapies to successfully treat iron-related disorders through the administration of probiotic bacteria that synthesize the metabolites involved in the regulation of iron uptake.^115^ From the first pioneer work until now, results concerning the effect of microbiota on iron metabolism have been similar. Even the secretion of lipocalin in liver in the case of anemia suggests that the host may use bacterial siderophores to replete its iron stores.^118^ This suggests that finding ways to modify the gut microbiota to modulate iron metabolism may be as important as studying the effect of iron on gut microbiota. The complexity of host and bacterial iron regulation and the cross-talk between the two is unpredictable, making it difficult to describe what happens during iron ingestion (whole meal, supplementation, and/or fortification). Considering the modifications in the effect of iron status on gut microbiota reported so far, further mechanistic studies are required to better understand their cross talk and to find other ways to fight iron deficiency related anemia, which is still a major challenge today.

## Data Availability

Not applicable

## List of abbreviations

ABC transporter: ATP-binding cassette transporter; ADP: adenosine di-phosphate; Apo-tf: transferrin without iron; ATP: adenosine tri-phosphate; Bcrp/Abcg2: breast cancer resistance protein/ATP-binding cassette subfamily G member 2; BMP: bone morphogenetic protein; CD91: cluster of differentiation 91; cDCs: conventional dendritic cells; DCYTB: Duodenal cytochrome B; Dmt1: Divalent metal transporter-1; DNA: Deoxyribonucleic acid; ECF: energy coupling factors; EDDA: ethylenediamine di-*O*-hydroxyphenyl acetic acid; EPO: erythropoietin; Feo: ferrous iron transport; FeoAB: ferrous transport fusion protein; Fe-S: iron-sulfur clusters; FLVCR: feline leukemia virus C receptor; Fur: ferric-uptake regulator protein; HAMP: hepcidin antimicrobial peptide; HCP1: Heme carrier protein 1; HFE: human homeostatic iron regulator protein; HFE2: hemochromatosis type 2 protein; HIF: hypoxia-inducible factors; HJV: Hepatic hemojuvelin protein; HOX-1: heme-oxygenase-1; HRG1: heme responsive gene; IL-1: interleukin-1; IL-6: interleukin-6; IM: inner membrane; IREs: iron responsive elements; IRPs: iron regulatory proteins JAK –STAT: Janus kinase signal transducer and activator of transcription signaling pathway; LIP: labile iron pool; LPS: lipopolysaccharides; mRNA: Messenger RNA; NaFeEDTA: ferric sodium ethylenediaminetetraacetate; Nramp: natural resistance associated macrophage protein transporters; OM: outer membrane; PBP: periplasmic binding proteins; PCBP: poly(rC)-binding protein; PCR: Polymerase chain reaction; rRNA: Ribosomal ribonucleic acid; SFB: Segmented filamentous bacteria; SMAD1, SMAD5, and SMAD8: *Drosphila* protein mothers against decapentaplegic (MAD) and of the SMA protein of *C. elegans*; Tf: transferrin; TfR1 and 2: transferrin receptor-1 and 2; TGGE: Temperature Gradient Gel Electrophoresis; ZIP-like transporters: zinc regulated transporters.
